# Assessing the Role of *Carotenoid Cleavage Dioxygenase 4* Homoeologs in Carotenoid Accumulation and Plant Growth in Tetraploid Wheat

**DOI:** 10.3389/fnut.2021.740286

**Published:** 2021-09-08

**Authors:** Shu Yu, Li Tian

**Affiliations:** Department of Plant Sciences, University of California, Davis, Davis, CA, United States

**Keywords:** wheat, carotenoid cleavage dioxygenase, *ccd4*, poltergeist-like, TILLING

## Abstract

The dietary needs of humans for provitamin A carotenoids arise from their inability to synthesize vitamin A *de novo*. To improve the status of this essential micronutrient, special attention has been given to biofortification of staple foods, such as wheat grains, which are consumed in large quantities but contain low levels of provitamin A carotenoids. However, there remains an unclear contribution of metabolic genes and homoeologs to the turnover of carotenoids in wheat grains. To better understand carotenoid catabolism in tetraploid wheat, Targeting Induced Local Lesions in Genomes (TILLING) mutants of *CCD4*, encoding a Carotenoid Cleavage Dioxygenase (CCD) that cleaves carotenoids into smaller apocarotenoid molecules, were isolated and characterized. Our analysis showed that *ccd4* mutations co-segregated with *Poltergeist-like* (*pll*) mutations in the TILLING mutants of A and B subgenomes, hence the *ccd-A4 pll-A, ccd-B4 pll-B*, and *ccd-A4 ccd-B4 pll-A pll-B* mutants were analyzed in this study. Carotenoid profiles are comparable in mature grains of the mutant and control plants, indicating that CCD4 homoeologs do not have a major impact on carotenoid accumulation in grains. However, the neoxanthin content was increased in leaves of *ccd-A4 ccd-B4 pll-A pll-B* relative to the control. In addition, four unidentified carotenoids showed a unique presence in leaves of *ccd-A4 ccd-B4 pll-A pll-B* plants. These results suggested that CCD4 homoeologs may contribute to the turnover of neoxanthin and the unidentified carotenoids in leaves. Interestingly, abnormal spike, grain, and seminal root phenotypes were also observed for *ccd-A4 pll-A, ccd-B4 pll-B*, and *ccd-A4 ccd-B4 pll-A pll-B* plants, suggesting that CCD4 and/or PLL homoeologs could function toward these traits. Overall, this study not only reveals the role of CCD4 in cleavage of carotenoids in leaves and grains, but also uncovers several critical growth traits that are controlled by CCD4, PLL, or the CCD4-PLL interaction.

## Introduction

Wheat is among the most widely cultivated and consumed staple food crops around the world. Wheat grains are a rich source of starch and proteins for human nutrition (1). In recent years, efforts have been directed toward enhancing the production of provitamin A carotenoids, particularly β-carotene, in wheat grains through breeding and biotechnology for improved vitamin A nutrition (2). However, provitamin A carotenoids produced in wheat grains may be subjected to degradation by carotenoid cleavage dioxygenases (CCDs) that cleave carotenoids (C_40_) into smaller apocarotenoid molecules (3). Therefore, it is imperative to better understand the activity and function of CCDs in wheat grains for achieving a high level of provitamin A carotenoid accumulation. Previous comparative analyses using CCDs from mouse [β-carotene 15,15′-monooxygenase-1 (BCMO1), BCMO2, and retinal pigment epithelium 65 (RPE65)], *Synechocystis* sp. PCC 6803 [lignostilbene dioxygenase (ACO)], and maize [9-*cis*-epoxycarotenoid dioxygenase (VP14)] revealed that CCDs from different kingdoms all contain four conserved histidine residues and additional acidic amino acids for binding of iron through hydrogen bonding, which is essential for the activity of the iron-dependent CCD enzymes (4).

Of the four CCDs (CCD1, CCD4, CCD7, and CCD8) identified in different plant species, CCD7 and CCD8 are dedicated to the biosynthesis of apocarotenoid phytohormones strigolactones, whereas CCD1 and CCD4 contribute to the production of various other apocarotenoid molecules in diverse tissues (5). Our recent study showed that *CCD1* and *CCD4* homoeologs are differentially expressed in tetraploid and hexaploid wheat grains (6). When assayed for enzyme activity using recombinant proteins, wheat CCD1 homoeologs, but not CCD4 homoeologs, converted β-carotene, lutein, and zeaxanthin to apocarotenoids, suggesting a role of CCD1 homoeologs in carotenoid degradation in wheat grains (6). However, genetic studies with *Arabidopsis* seeds demonstrated that *At*CCD4 plays a major role in degradation of β-carotene during desiccation of *Arabidopsis* seeds, though *At*CCD4 exhibited low *in vitro* enzyme activity toward β-carotene (7). Additionally, total carotenoids in chrysanthemum petals and the violaxanthin content in potato tubers were increased when *CCD4* expression was downregulated via RNA interference (RNAi) in these tissues, suggesting its role in carotenoid cleavage *in planta* (8, 9). Taking into consideration these reports from wheat and other plants, it remains to be determined whether CCD4 could catalyze carotenoid cleavage reactions in wheat, which could be interrogated through genetic manipulation of CCD4 enzyme activity or gene expression. Emerging evidence has suggested the role of apocarotenoid signaling molecules, other than strigolactones and abscisic acid [ABA; produced by nine-*cis*-epoxycarotenoid dioxygenases (NCEDs)], in controlling plant growth, development, and interactions with the environment (5). Therefore, it will be important to also understand the function of apocarotenoid molecules generated by CCD4 in wheat.

**Figure 1:**
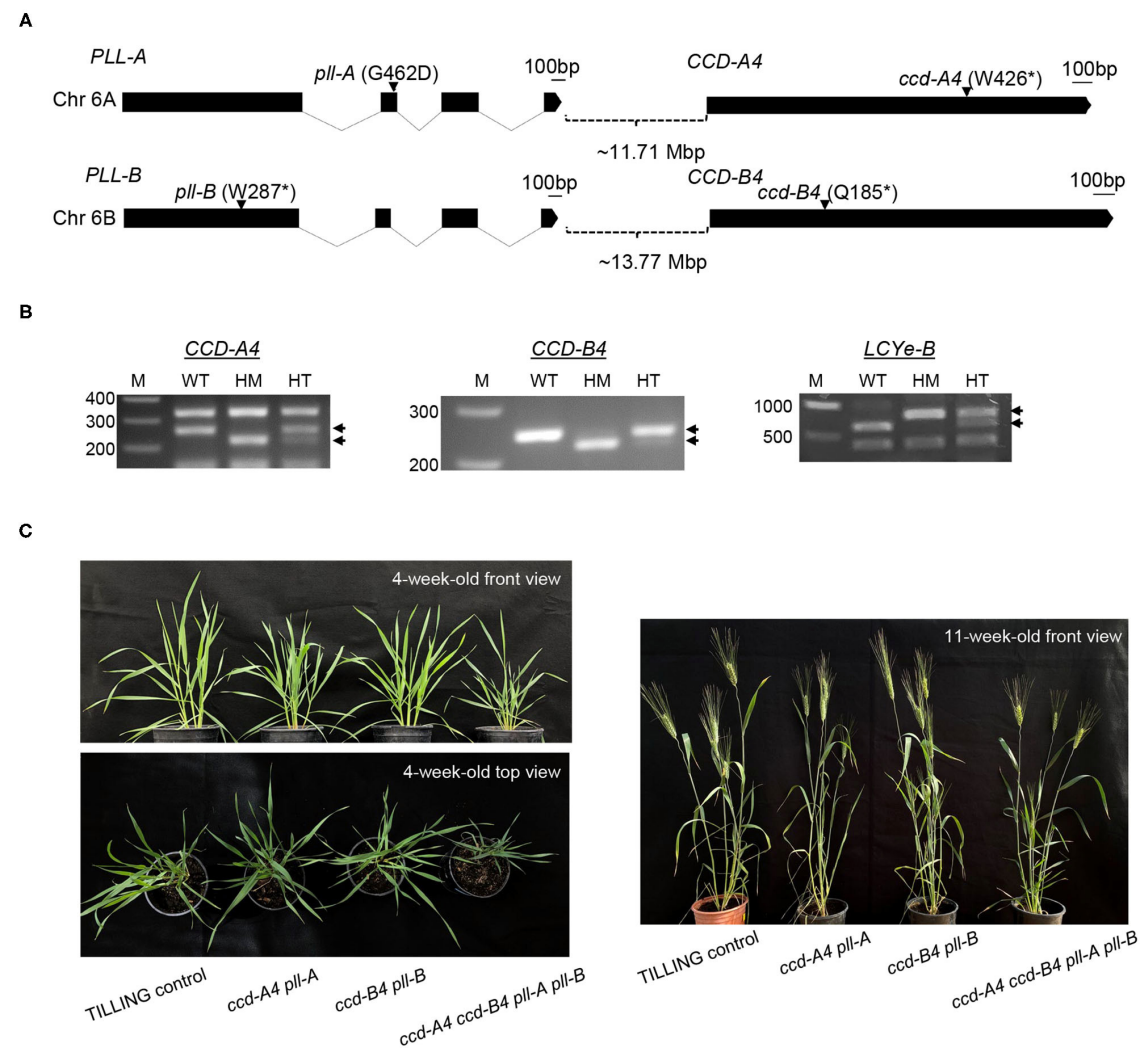
Tetraploid wheat TILLING mutants of *CCD-A4, CCD-B4, PLL-A*, and *PLL-B*. **(A)** Exon-intron diagrams of *CCD-A4, CCD-B4, PLL-A*, and *PLL-B*. Exons and introns are shown by black boxes and solid lines, respectively. The black bar denotes 100 bp. Locations of mutations are indicated with arrows. Mutant alleles of *CCD4* and *PLL* homoeologs are linked and co-segregated in progenies of *ccd-A4* and *ccd-B4* mutants. Chr, chromosome; Mbp, megabase pair; *stop codon. **(B)** Cleaved Amplified Polymorphic Sequences (CAPS) and derived CAPS (dCAPS) markers for genotyping of wild-type and mutant *CCD-A4, CCD-B4*, and *LCYe-B* alleles. The PCR products were digested with HphI (*CCD-A4* primer pair), RsaI (*CCD-B4* primer pair), and DdeI (*LCYe-B* primer pair). Diagnostic bands for each marker are indicated with arrows. WT, homozygous wild type for the target gene; HM, homozygous mutant; HT, heterozygous mutant; M, DNA size marker. **(C)** Images of 4-week-old and 11-week-old TILLING control as well as *ccd-A4 pll-A, ccd-B4 pll-B*, and *ccd-A4 ccd-B4 pll-A pll-B* mutant plants.

To investigate the function of CCD4 homoeologs in carotenoid metabolism and plant growth in tetraploid wheat, we isolated Targeting Induced Local Lesions in Genomes (TILLING) mutants of *CCD4* homoeologs from a tetraploid wheat mutant library in this study. *CCD4* is located near a gene annotated as *Poltergeist-like* (*PLL*) on chromosome 6 of tetraploid wheat (Figure 1A). PLL and its homolog Poltergeist (POL) belong to the protein phosphatase type 2C (PP2C) family and contain metal ion-interacting domains with highly conserved amino acid residues (10). The *Arabidopsis* POL and PLL1 were shown to function in maintenance and differentiation of stem cells as well as additional developmental pathways such as formation of the central vasculature and embryo development (10–15). Although the *Arabidopsis pol* and *pll1* single mutants only displayed weak developmental phenotypes relative to wild-type plants (10, 15), the *pol pll1* double mutants were seedling lethal, indicating that both POL and PLL1 are crucial for plant development (13). Besides PLL1, there are four additional PLLs in *Arabidopsis*: PLL2-PLL5. While the mutant analysis demonstrated that PLL4 and PLL5 regulate leaf morphology, *pll2* and *pll3* mutants did not exhibit any distinguishable growth phenotypes when compared to wild-type plants, suggesting that PLL2 and PLL3 may not play a developmental role in *Arabidopsis* (12).

Our analysis showed that the *ccd4* TILLING mutant lines isolated in this study also contain mutations in *PLL* and that the *ccd4* mutations are linked to the *pll* mutations in both A and B subgenomes in the backcrossed (BCed) progenies. Because *CCD4* and *PLL* are closely located in the same chromosomal region, there is a low frequency of recombination between the two genes during meiosis and they are inherited together in the next generation. As such, the double (*ccd-A4 pll-A* and *ccd-B4 pll-B*) and quadruple (*ccd-A4 ccd-B4 pll-A pll-B*) mutants were analyzed in this study for biochemical and growth phenotypes and agronomic traits.

## Materials and Methods

### Plant Growth and Tissue Collection

Wheat seeds were surface-sterilized using 1% (w/v) sodium hypochlorite solution containing 0.1% (v/v) Triton X-100, and rinsed with running water for at least 3 times. The sterilized seeds were placed on two layers of damp germination paper (Hoffman Manufacturing, Inc., Corvallis, OR) in a petri dish and stored at 4°C for 3 d to synchronize germination. The cold-treated seeds were subsequently moved to room temperature (~22°C) and germinated in the dark for 2–4 d to allow root development. The seedlings were then transplanted in soil and grown in a climate-controlled greenhouse (~22°C) under long-day conditions (16-h light/8-h dark). For leaf carotenoid and gene expression analyses, the fourth leaf counted from the top on the primary tiller of 4-week-old plants was collected, frozen in liquid nitrogen, and stored at −80°C until analysis. For grain carotenoid, spike, and grain yield analyses, spikes were collected from wheat plants at the harvest-ready stage and dried at room temperature (~22°C) for 1 week before the measurements. Grains were hand-cleaned to remove dry husks, and those harvested from the same plant were pooled and considered as one biological replicate. For grain carotenoid analysis, 25 whole grains were randomly sampled from each biological replicate and used for extraction.

### TILLING Mutant Screening and Crossing

Mutants of *CCD-A4* and *CCD-B4* (i.e., *ccd-A4* and *ccd-B4*) were identified from the exome-sequenced TILLING mutant library of tetraploid wheat cv. Kronos (https://dubcovskylab.ucdavis.edu/wheat_blast) using the respective DNA sequences as queries. To reduce the additional mutations caused by ethyl methanesulfonate (EMS), the M_4_ *ccd-A4* (line T4-0842) and *ccd-B4* (line T4-3179) mutants were BCed to the wild-type parental plant Kronos for one generation. The BC_1_ *ccd-A4* and *ccd-B4* mutant plants were then intercrossed and the heterozygous progenies harboring *ccd-A4* and *ccd-B4* mutant alleles were chosen for self-pollination. From the segregating population of the self-pollinated plants, mutants that are homozygous for *ccd-A4* or *ccd-B4*, or both *ccd-A4* and *ccd-B4* were selected. TILLING controls are plants containing wild-type *CCD-A4* and *CCD-B4* alleles, and have a mutational load similar to that of *ccd-A4, ccd-B4*, and *ccd-A4 ccd-B4* mutants; TILLING controls were also selected from the segregating population. Cleaved Amplified Polymorphic Sequences (CAPS) and derived CAPS (dCAPS) markers were designed for *CCD-A4* and *CCD-B4* and used in the genotyping analysis (Supplementary Table 1).

A mutated *lycopene* ε*-cyclase-B* (*LCYe-B*) allele is also present in the M_4_ *ccd-B4* plant (line T4-3179), but segregated independently from the *ccd-B4* mutation when BCed to the wild-type Kronos plants. TILLING control and the homozygous BC_1_ *ccd-A4, ccd-B4*, and *ccd-A4 ccd-B4* mutants were inspected for *LCYe-B* alleles using a CAPS marker (Supplementary Table 1); all of the above-mentioned genotypes contain the wild-type *LCYe-B* allele and were used in this study. The *PLL* homoeologs were amplified from the *ccd-A4, ccd-B4, ccd-A4 ccd-B4* mutants and TILLING control and subjected to DNA sequencing (primer pairs are listed in Supplementary Table 2). This confirmed that the *ccd-A4* mutant harbored *pll-A* and *PLL-B* alleles, the *ccd-B4* mutant contained *PLL-A* and *pll-B* alleles, and the *ccd-A4 ccd-B4* mutant carried *pll-A* and *pll-B* alleles. TILLING control plants were verified to possess only wild-type *CCD-A4, CCD-B4, PLL-A*, and *PLL-B* alleles. Because the *ccd4* mutants also contain homozygous *pll* mutations, they were therefore designated the *ccd-A4 pll-A, ccd-B4 pll-B*, and *ccd-A4 ccd-B4 pll-A pll-B* mutants.

### Multiple Sequence Alignment

The GenBank accession numbers of the selected proteins are: *Synechocystis* sp. PCC 6803 lignostilbene dioxygenase, BAA18428; maize VP14, AAB62181; tetraploid wheat CCD-A4, KU975448; tetraploid wheat CCD-B4, KU975449; *At*PLL1, NP_181078; *At*POL, NP_850463; tetraploid wheat PLL-A, XP_037447847 (this sequence from *Triticum dicoccoides* is identical to PLL-A from *T. turgidum*); tetraploid wheat PLL-B, XP_037453069 (this sequence from *T. dicoccoides* is identical to PLL-B from *T. turgidum*). The protein sequences were aligned using Clustal Omega (16) and the alignment diagrams were prepared using BoxShade (https://embnet.vital-it.ch/software/BOX_form.html).

### Carotenoid Analysis

Leaves and mature whole grains were ground into fine powder in liquid nitrogen using a mortar and pestle. Total carotenoids were extracted from ~50 mg leaves and ~300 mg whole grain flour using the methods described in (6) and (17), respectively. Carotenoids extracted from grain flour were saponified using 2 M KOH [dissolved in methanol containing 0.01% (w/v) butylated hydroxytoluene]. Following saponification in dark for 30 min, equal volumes of diethyl ether and H_2_O were added for phase separation. Re-extraction of carotenoids from the water phase, pooling and washing the diethyl ether layers, and drying and re-dissolving of carotenoid residues were carried out as described (17). Ten microliter of leaf or grain carotenoid extract (resuspended in ethyl acetate) was injected on a reverse-phase HPLC column and analyzed using a previously established gradient (18).

### Gene Expression Analysis

Total RNA was extracted from ~50 mg of ground leaves using a cetyltrimethylammonium bromide (CTAB)-based method (19). After treatment with RNase-free DNase I (Fermentas, Glen Burnie, MD), reverse transcription was carried out using the iScript™ Advanced cDNA Synthesis Kit (BioRad, Hercules, CA) following the manufacturer's instructions. For each qPCR reaction, 0.1 μl first-strand cDNA (equivalent to 6.25 ng total RNA) was used as template for amplification with the iTaq™ Universal SYBR® Green Supermix (BioRad). Primers used for the real-time qPCR analysis of *CCD-A4* and *CCD-B4*, as well as the reference genes *Ta2291* and *Ta54227*, were as previously described (6).

### Measurement of Plant Growth Traits

For analysis of spike and grain traits, nine plants of TILLING control or each of the mutant genotypes were assessed for the total number of spikes per plant. The number of spikelets on each spike was counted and averaged for all spikes in the same plant to be considered as one biological replicate. To determine the length of primary spike, the spike on the first tiller of the plant was measured for the distance between the base and the top of the spike without the awn. The total number of grains was counted for each plant. Grain weight was then calculated by dividing the weight of all grains harvested from a plant by the number of grains for that plant. Images of grains were captured using an Epson V600 scanner (Epson, Los Alamitos, CA) with the crease side down. The grain length and width were determined using ImageJ (20) and averaged for all grains collected from each plant.

For analysis of seminal root traits, one sterilized and cold-treated wheat seed was placed in between a piece of germination paper and a clear sheet protector with the crease side of the seed oriented toward the germination paper and the embryo end pointing downwards. The seeds were grown vertically at the room temperature (~22°C) either entirely in the dark or under long-day conditions (16-h light/8-h dark) with a light intensity of 120 μmol m^−2^ s^−1^. After germinating at room temperature for 3 d, the seedlings were scanned using an Epson V600 scanner (Epson) and images saved as TIFF files. Coleoptiles and seminal roots were hand-traced in the images and the trait parameters were determined using ImageJ (20).

### Statistical Analysis

One-way Analysis of Variation (ANOVA) followed by Tukey's Honestly Significant Difference test were performed for the carotenoid quantification, gene expression, spike, grain, and seminal root data using JMP (SAS Institute, Cary, NC).

## Results

### The *ccd4* TILLING Mutants Used in This Study Do Not Contain Mutations in Other Carotenoid Metabolic Gene Homoeologs, but Carry *pll-A* and *pll-B* Mutations

By searching an exome-sequenced tetraploid wheat TILLING mutant library (21), 78 and 109 lines were identified that contain mutations in the open reading frame (ORF) of *CCD-A4* and *CCD-B4*, respectively. Of these mutations, three led to premature stop codons in CCD-A4: W426^*^ in line T4-0842, W441^*^ in line T4-2477, and Q628^*^ in line T4-0594, and one in CCD-B4: Q181^*^ in line T4-3179. Lines T4-0842 (W426^*^) and T4-3179 (Q181^*^) were used in this study and designated as *ccd-A4* and *ccd-B4*. The W426^*^ mutation in *ccd-A4* truncated 203 amino acids out of the 628 amino acids of CCD-A4. The Q181^*^ mutation in *ccd-B4* led to a CCD-B4 protein missing 452 amino acids from the C-terminus (Figure 1A). These truncated CCD-A4 and CCD-B4 proteins do not contain the entire four-His and three-Glu iron coordination system essential for CCD enzyme activity, suggesting that they are loss-of-function mutations (Supplementary Figure 1) (22).

The exome sequences of *ccd-A4* and *ccd-B4* were also analyzed for possible mutations in other carotenoid metabolic genes, including *lycopene* β*-cyclase* (*LCYb*), *LCYe*, β*-carotene hydroxylase 1* (*HYD1*), *HYD2*, and *CCD1*. The absence of mutations was verified for all of these gene homoeologs (i.e., both A and B subgenomes) except for *LCYe-B*; *ccd-B4* (line T4-3179 at the M_4_ generation) also contained a mutation in *LCYe-B*. The *ccd-A4* and *ccd-B4* mutants were each BCed to the wild-type parental line Kronos for one generation. The *LCYe-B/lcye-B* (chromosome 3) and *CCD-B4/ccd-B4* (chromosome 6) alleles segregated independently during BC as they are located on different chromosomes. We confirmed that all the *ccd4* mutants used in this study (at the BC_1_ generation) only contain the wild-type *LCYe-B* alleles according to genotyping analysis using a CAPS marker (Figure 1B). The BC_1_ *ccd-A4* and *ccd-B4* mutants were crossed and the progenies genotyped to select the *ccd-A4 ccd-B4* double mutants using CAPS and dCAPS markers (Figure 1B).

Genes surrounding *CCD4* on chromosome 6 were also inspected for mutations using the exome sequences of *ccd-A4* and *ccd-B4*. Both *ccd-A4* and *ccd-B4* mutants carried mutated *PLL* alleles (Figure 1A) and we confirmed that *pll* mutations were linked with *ccd4* mutations in the BC_1_ *ccd4* mutants by DNA sequencing (data not shown). The mutation of *PLL-A* led to an amino acid substitution (G462D) and the mutation of *PLL-B* led to a truncated PLL-B (W287^*^) protein (Figure 1A). These mutated proteins miss either a critical amino acid in a highly conserved region (pll-A) or essential functional components of protein phosphatases (pll-B), suggesting that they are likely loss-of-function mutations (Supplementary Figure 2). Taken together from the mutant analysis, the *ccd-A4, ccd-B4*, and *ccd-A4 ccd-B4* mutants used in this study are indeed *ccd-A4 pll-A, ccd-B4 pll-B*, and *ccd-A4 ccd-B4 pll-A pll-B*, respectively. In addition to these mutant genotypes, TILLING control plants that are wild-type for the *CCD-A4, CCD-B4, PLL-A*, and *PLL-B* alleles, and carry a similar mutational load as the mutants were analyzed in parallel with the mutants.

### Mutations of *CCD-A4* and *CCD-B4* Are Associated With Altered Carotenoid Profiles and Varied Expression of *CCD4* Homoeologs in Leaves

**Figure 2:**
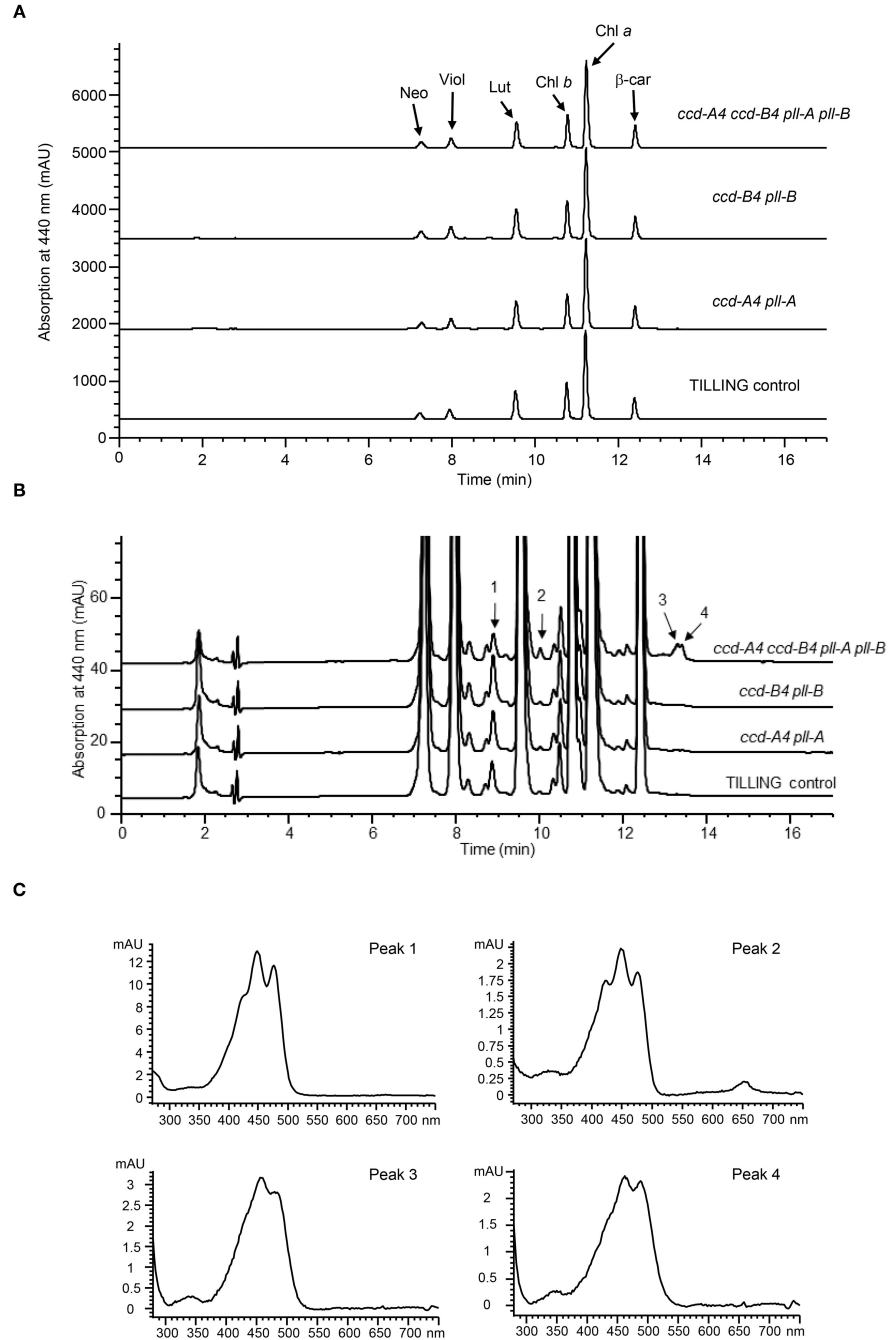
HPLC analysis of carotenoid profiles in leaves of TILLING control and mutant plants. **(A)** HPLC chromatograms of TILLING control, *ccd-A4 pll-A, ccd-B4 pll-B*, and *ccd-A4 ccd-B4 pll-A pll-B*. Neo, neoxanthin; Viol, violaxanthin; Lut, lutein; Chl *b*, chlorophyll *b*; Chl *a*, chlorophyll *a*; β-car, β-carotene. **(B)** A zoomed view of HPLC chromatograms showing peaks that are differentially accumulated in *ccd-A4 ccd-B4 pll-A pll-B* and TILLING control. **(C)** Absorption spectra of peaks 1–4.

To understand the impact of *ccd4* mutations on carotenoid accumulation, total carotenoids were extracted from leaves and mature whole grains of TILLING control and the mutant plants and analyzed on high performance liquid chromatography (HPLC) (Figure 2; Tables 1, 2). In leaves, a small but significant increase was observed in neoxanthin levels in *ccd-B4 pll-B* and *ccd-A4 ccd-B4 pll-A pll-B*, in violaxanthin levels in *ccd-A4 pll-A* and *ccd-B4 pll-B*, in lutein levels in *ccd-B4 pll-B*, and in total carotenoids in *ccd-B4 pll-B* and *ccd-A4 ccd-B4 pll-A pll-B*, relative to TILLING control (Table 1). There was no significant change in the β-carotene content between TILLING control and the mutants (Table 1). Although accumulated at very low levels, two unidentified peaks eluted at 8.89 min (peak 1) and 10.01 min (peak 2) showed differential accumulation only in *ccd-A4 ccd-B4 pll-A pll-B* (Figure 2B; Supplementary Table 1). Additionally, two unidentified peaks eluted at 13.28 min (peak 3) and 13.39 min (peak 4) were present in the leaf carotenoid extracts of *ccd-A4 ccd-B4 pll-A pll-B* but absent in other genotypes (Figure 2B; Supplementary Table 3). Peaks 1–4 appear to be carotenoid molecules as they possess the characteristic three-peak absorption profiles for carotenoids (Figure 2C). In mature whole grains, lutein, β-carotene, and total carotenoids were not significantly different among TILLING control and the mutant genotypes (Table 2).

**Table 1:** Carotenoids (mmol mol^−1^ chlorophylls *a* + *b*) in leaves of 4-week-old TILLING control and mutant plants.

**Genotype**	**Neoxanthin**	**Violaxanthin**	**β-carotene**	**Lutein**	**Total**
TILLING control	20.36 ± 0.69^a^	24.19 ± 1.21^a^	47.31 ± 2.28^a^	70.25 ± 1.73^a^	162.11 ± 3.02^a^
*ccd-A4 pll-A*	21.48 ± 1.01^ab^	26.78 ± 1.61^b^	45.33 ± 2.67^a^	72.38 ± 0.92^ab^	165.97 ± 3.84^ab^
*ccd-B4 pll-B*	21.78 ± 0.98^b^	27.13 ± 1.81^b^	47.90 ± 4.16^a^	73.15 ± 1.17^b^	165.95 ± 1.86^b^
*ccd-A4 ccd-B4 pll-A pll-B*	22.48 ± 0.52^b^	26.14 ± 0.91^ab^	47.77 ± 1.11^a^	70.87 ± 1.09^ab^	167.25 ± 3.37^b^

*Data presented are mean ± standard deviation of 4–8 biological replicates. Different letters indicate statistically significant differences (P < 0.05) within a column*.

**Table 2:** Carotenoids (nmol g^−1^ flour) in mature whole grains of TILLING control and mutant plants.

**Genotype**	**Lutein**	**β-carotene**	**Total**
TILLING control	4.94 ± 1.14^a^	0.21 ± 0.05^a^	5.15 ± 1.16^a^
*ccd-A4 pll-A*	5.20 ± 1.56^a^	0.20 ± 0.04^a^	5.40 ± 1.53^a^
*ccd-B4 pll-B*	3.98 ± 1.53^a^	0.21 ± 0.06^a^	4.18 ± 1.58^a^
*ccd-A4 ccd-B4 pll-A pll-B*	5.30 ± 1.27^a^	0.20 ± 0.05^a^	5.50 ± 1.26^a^

*Data presented are mean ± standard deviation of 5-6 biological replicates. Different letters indicate statistically significant differences (P < 0.05) within a column*.

**Figure 3:**
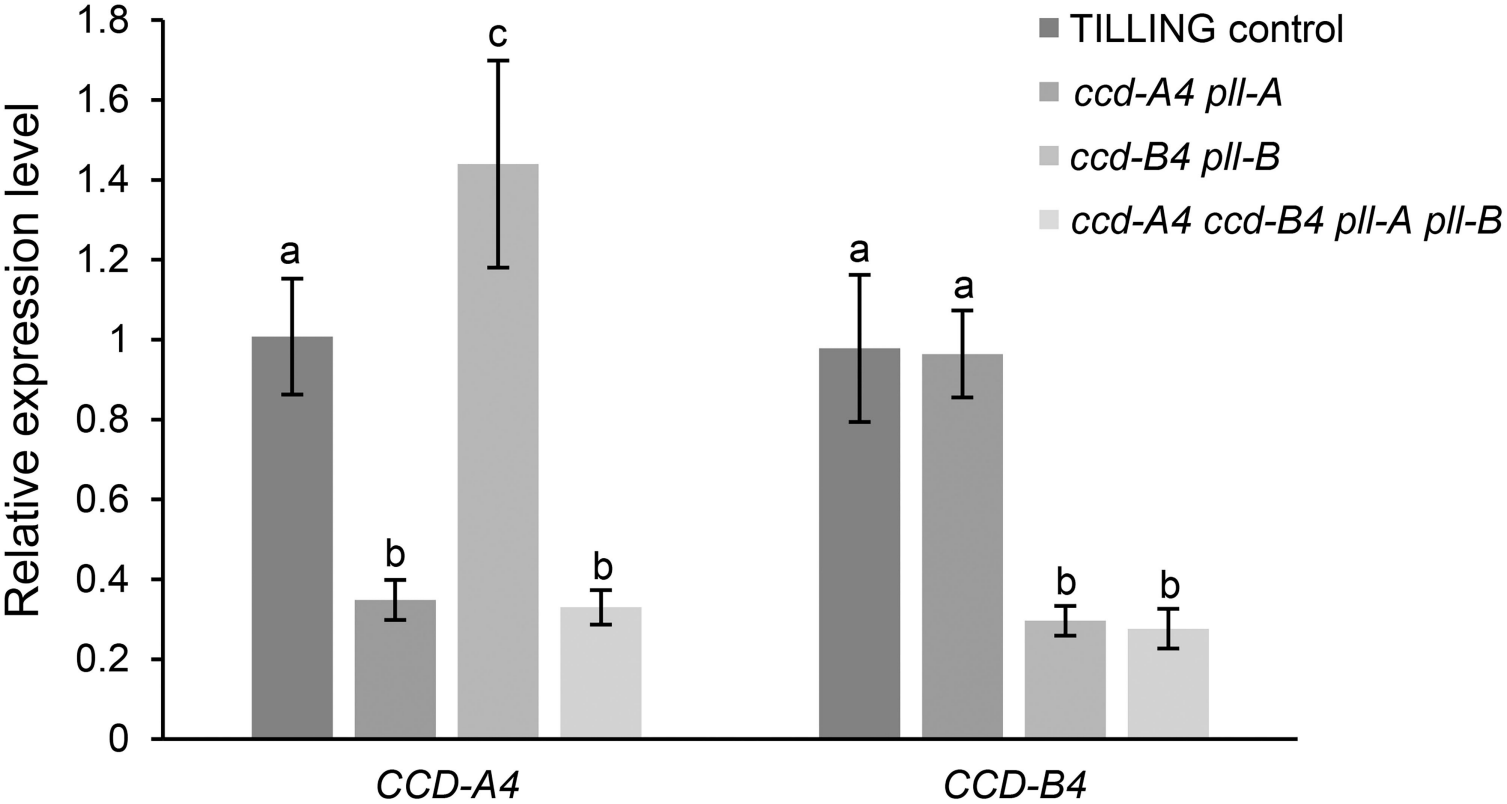
Expression levels of *CCD-A4* and *CCD-B4* in leaves of TILLING control and mutant plants. Data presented are mean ± standard deviation of 4-8 biological replicates. Different letters indicate significantly different expression (*P* < 0.05) for *CCD-A4* or *CCD-B4* in different genotypes.

To examine whether the mutation of one *CCD4* homoeolog may cause changes in the expression of the other *CCD4* homoeolog, transcript levels of *CCD-A4* and *CCD-B4* in leaves of the mutant and TILLING control plants were determined using real-time qPCR (Figure 3). Expression levels of the mutated *CCD-A4* and *CCD-B4* alleles were only about 30% of the wild-type alleles in TILLING control, suggesting that the mutations not only led to premature stop codons, but also reduced the stability of the respective mRNAs (Figure 3). While the expression of *CCD-B4* in *ccd-A4 pll-A* maintained at a level comparable to that in TILLING control, the expression of *CCD-A4* was slightly higher in *ccd-B4 pll-B* than TILLING control, suggesting that the *ccd-B4* mutation may induce *CCD-A4* expression (Figure 3).

### The *ccd-A4 ccd-B4 pll-A pll-B* Mutant Differed Greatly in Spike and Grain Phenotypes

**Figure 4:**
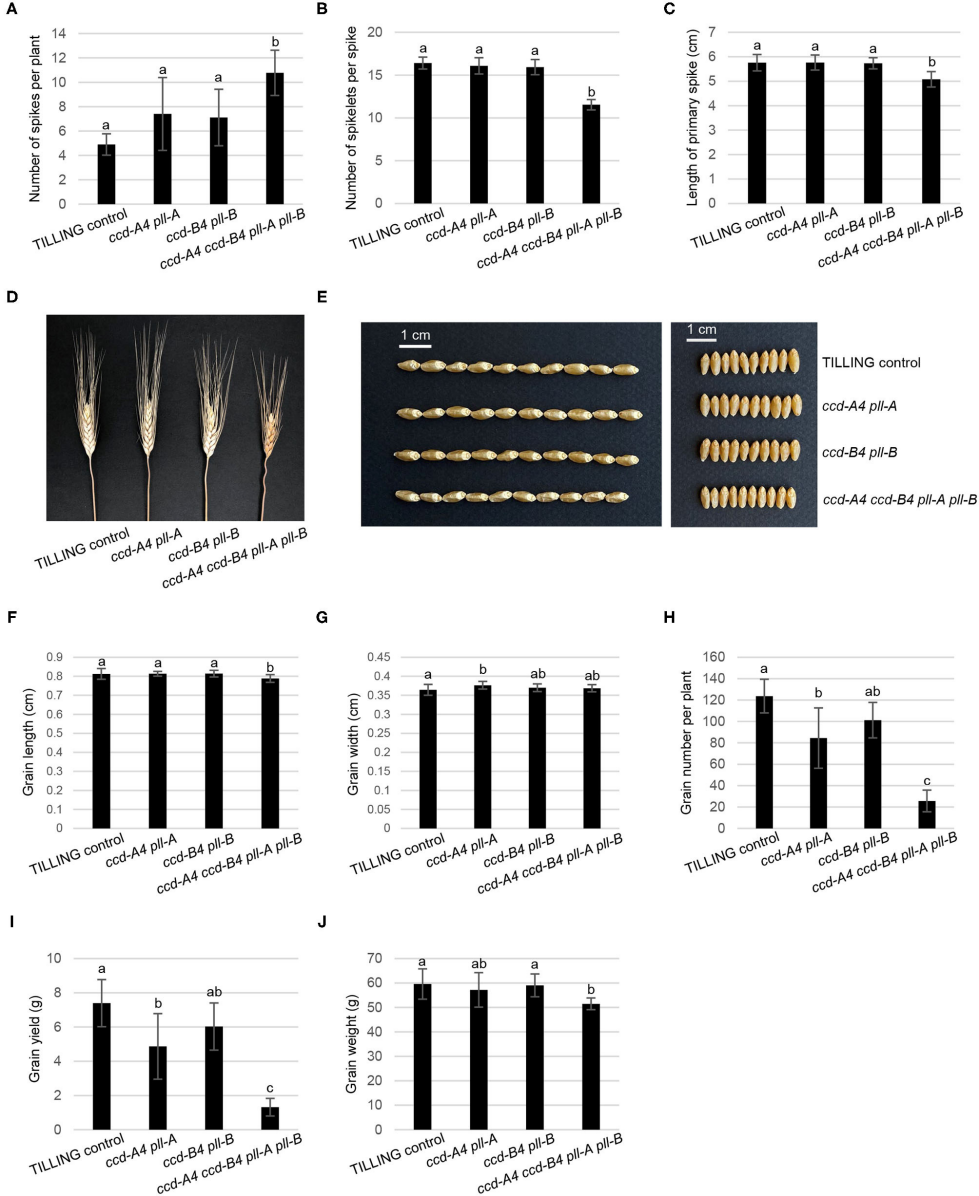
Spike and grain phenotyping of TILLING control and mutant plants. **(A)** Number of spikes per plant. **(B)** Number of spikelets per spike. **(C)** Length of primary spike. **(D)** Image of TILLING control and mutant spikes. **(E)** Images of TILLING control and mutant grains. **(F)** Grain length. **(G)** Grain width. **(H)** Grain number per plant. **(I)** Grain yield. **(J)** Grain weight. The mean ± standard deviation of 9 biological replicates are shown. Different letters denote significantly different (*P* < 0.05) for each spike or grain trait.

When grown in the greenhouse under long-day conditions, *ccd-A4 ccd-B4 pll-A pll-B* plants were apparently smaller than *ccd-A4 pll-A, ccd-B4 pll-B*, and TILLING control plants at both early (4-week-old, prior to the emergence of spikes) and late (11-week-old, physiological maturity) developmental stages (Figure 1C). To evaluate the effect of *ccd4* and *pll* mutations on plant performance and grain quality, several agronomic traits related to grain yield were determined that include the number of spikes per plant, number of spikelets per spike, grain number per plant, grain weight, and grain size (length and width) (Figure 4). When evaluated using tissues collected at the harvest-ready stage of mature plants, *ccd-A4 ccd-B4 pll-A pll-B* has 120% more spikes per plant than TILLING control, and ~50% more spikes than *ccd-A4 pll-A* and *ccd-B4 pll-B* (Figure 4A). However, *ccd-A4 ccd-B4 pll-A pll-B* has 40% less spikelets per spike than TILLING control, and ~30% less spikelets per spike than *ccd-A4 pll-A* and *ccd-B4 pll-B* (Figure 4B). As a result of the reduced number of spikelets on the spike, the primary spike in *ccd-A4 ccd-B4 pll-A pll-B* was ~12% shorter compared with other genotypes (Figures 4C,D).

Besides the visibly different spike phenotypes (Figure 4D), grains harvested from *ccd-A4 ccd-B4 pll-A pll-B* appeared to be slightly smaller than those from TILLING control and other mutants (Figure 4E). When quantified, *ccd-A4 ccd-B4 pll-A pll-B* grains were 3% shorter than TILLING control and other mutants, whereas grain width was comparable for grains of all genotypes (Figures 4F,G). On the other hand, the number of grains per plant and grain yield were more drastically reduced for *ccd-A4 ccd-B4 pll-A pll-B* relative to TILLING control, *ccd-A4 pll-A*, and *ccd-B4 pll-B*, suggesting that the *ccd-A4 ccd-B4 pll-A pll-B* mutant has reduced fertility (Figures 4H,I). Indeed, we observed that spikes on the lateral tillers of *ccd-A4 ccd-B4 pll-A pll-B* were sterile despite its large number of lateral tillers (data not shown). Consistent with the relatively smaller grains, the average weight of grains was also decreased by ~11% in *ccd-A4 ccd-B4 pll-A pll-B* compared to TILLING control and other mutants (Figure 4J).

### The *ccd-A4 ccd-B4 pll-A pll-B* Mutant Exhibited Distinct Seminal Root Phenotypes in Seedlings Grown in the Dark and Under Long-Day Conditions

**Figure 5:**
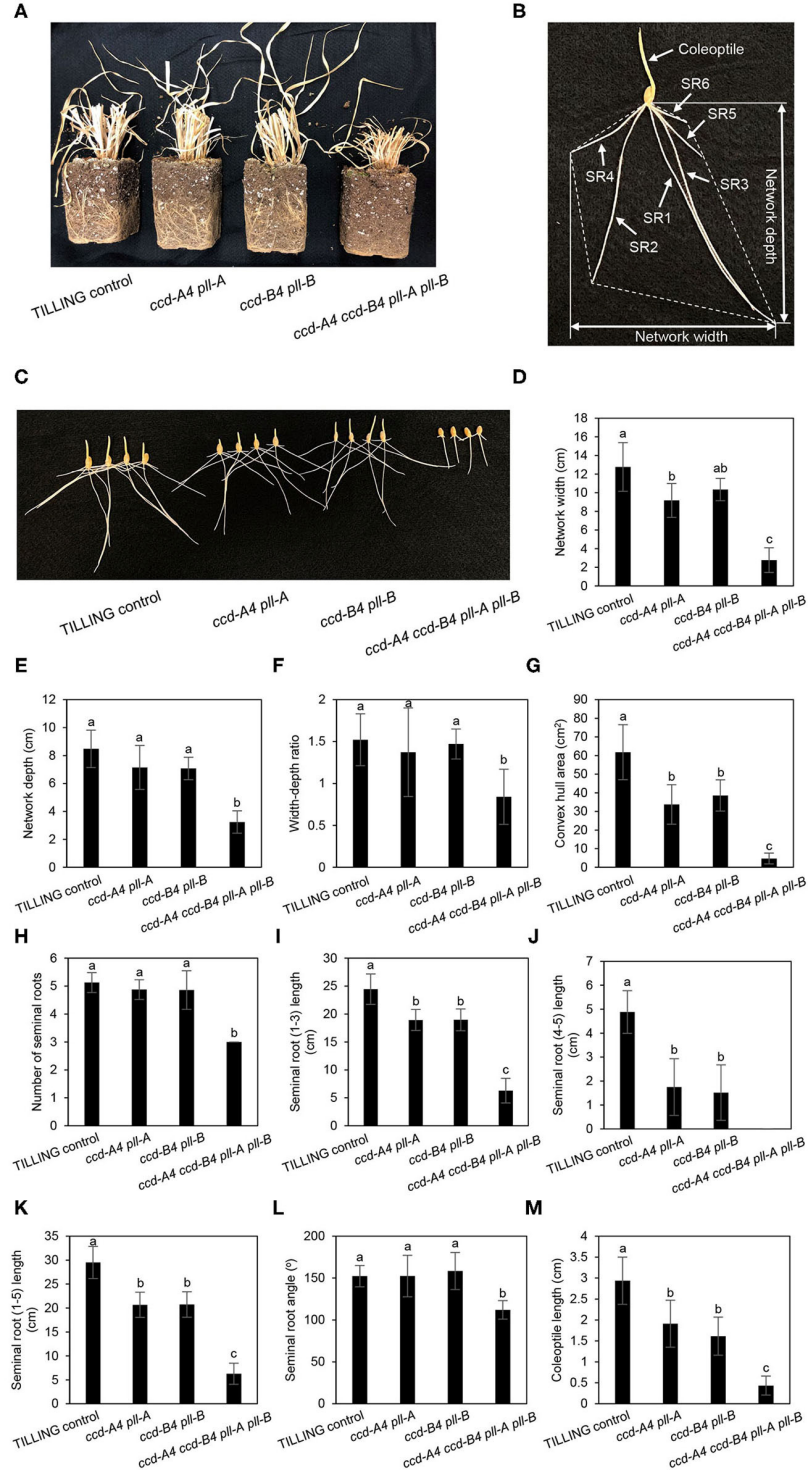
Seminal root phenotyping of TILLING control and mutant seedlings grown in the dark. **(A)** Overview of root growth for TILLING control and mutant plants at harvest. **(B)** Diagram showing analysis of wheat seminal root parameters. The convex hull area refers to the smallest area covered by a convex polygon containing the root system and is delineated with dotted lines. SR, seminal root. **(C)** Image of 3-day-old seedlings grown in the dark. **(D–L)** Represent different seminal root traits. **(M)** Coleoptile length. The mean ± standard deviation of 11 seedlings are shown. Different letters denote significantly different (*P* < 0.05) for each seminal root trait or coleoptile length.

**Figure 6:**
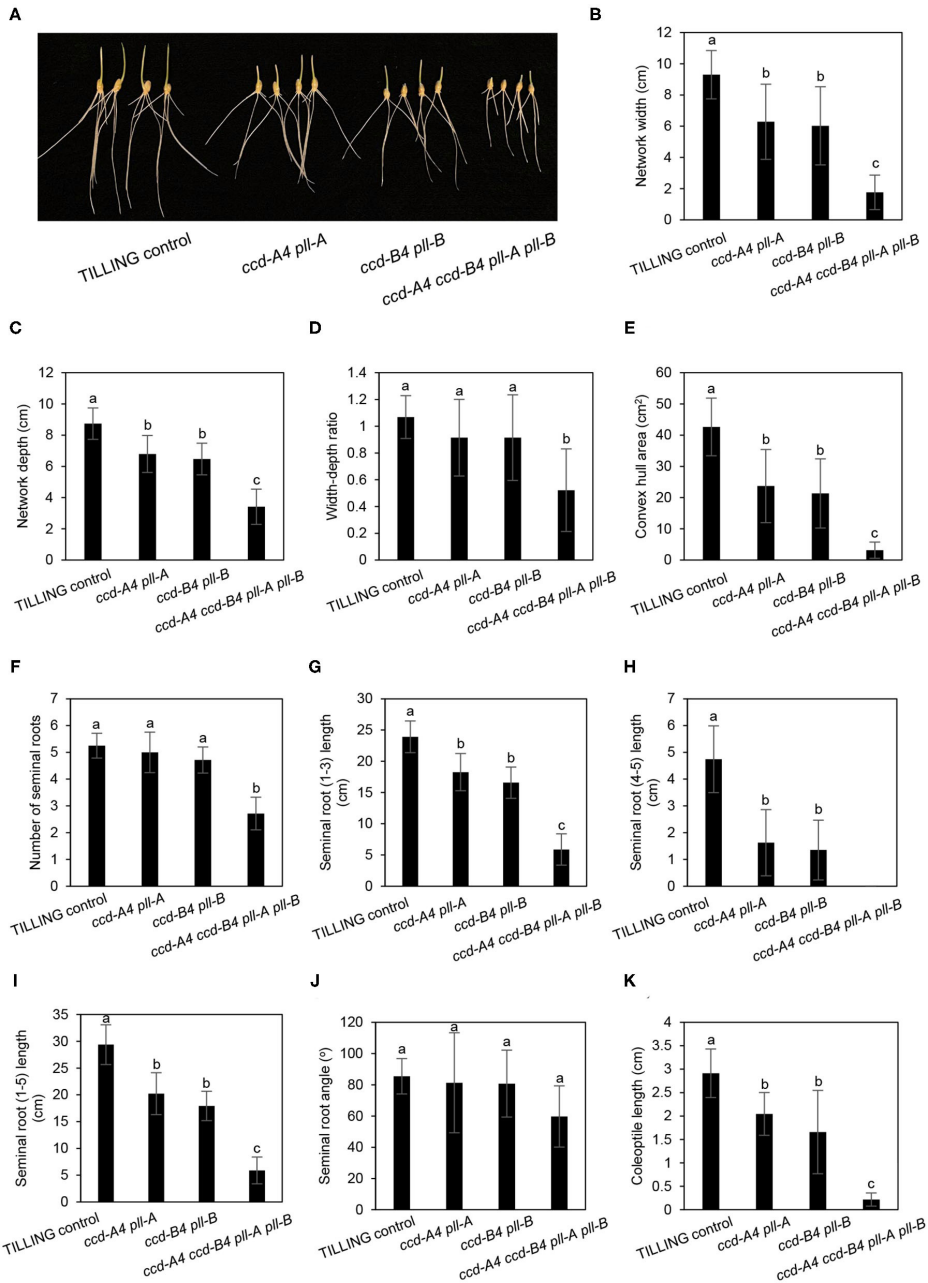
Seminal root phenotyping of TILLING control and mutant seedlings grown under long-day conditions. **(A)** Image of 3-day-old seedlings grown under long-day conditions. **(B–J)** Represent different seminal root traits. **(K)** Coleoptile length. The mean ± standard deviation of 11 seedlings are shown. Different letters denote significantly different (*P* < 0.05) for each seminal root trait or coleoptile length.

In mature plants at harvest, *ccd-A4 ccd-B4 pll-A pll-B* plants possess a largely reduced root volume, which may lead to less biomass production and yield as the root system is responsible for taking up water and mineral nutrients from the soil (Figure 5A). Wheat plants contain both seminal roots that develop from the radical and nodal (aka. crown or adventitious) roots that develop from nodes of the stem. To understand whether the *ccd4* and/or *pll* mutations affect root growth, seminal root traits of seedlings were analyzed for 3-day-old seedlings grown in dark or long-day conditions, as both lighting schemes have been used in wheat seed germination (Figures 5, 6). A total of seven seminal root traits were evaluated, including network width, network depth, network width to depth ratio, convex hull area (the area of the smallest convex polygon to enclose the root system), number of seminal roots, seminal root length, and seminal root angle (the angle between the outermost seminal roots). Coleoptile lengths of the seedlings were also measured.

For dark-grown seedlings, the network width, depth, width to depth ratio, convex hull area, and angle of seminal roots were 26–80% reduced in *ccd-A4 ccd-B4 pll-A pll-B* relative to TILLING control (Figures 5D–G). While the reduced seminal root angle was due to a lack of seminal roots 4 and 5 in *ccd-A4 ccd-B4 pll-A pll-B* seedlings after 3 d of germination, the reduced network depth and width resulted from a slower growth of seminal roots 1 (depth) and 2 and 3 (width) in this mutant (Figures 5D,E,L). In fact, the emergence of the fourth seminal root was only observed in very few 11-day-old *ccd-A4 ccd-B4 pll-A pll-B* seedlings, indicating that the initiation of the fourth seminal root was severely delayed in this mutant (Supplementary Figure 3). The combined absence of seminal roots 4 and 5 and a slow growth of seminal roots 1–3 also led to the reduced network width-depth ratio and convex hull area in 3-day-old *ccd-A4 ccd-B4 pll-A pll-B* seedlings. The total length of seminal roots #1-3 of *ccd-A4 ccd-B4 pll-A pll-B* was 75% shorter than that of TILLING control (Figure 5I). Although the 3-day-old *ccd-A4 pll-A* and *ccd-B4 pll-B* seedlings possessed 5 seminal roots, their total seminal root lengths were ~30% shorter than TILLING control (Figures 5I–K). The coleoptile lengths correlated with seminal root lengths (seminal roots 1–5) in all mutant genotypes with *ccd-A4 pll-A* and *ccd-B4 pll-B* showing 35–45% reduced length, and *ccd-A4 ccd-B4 pll-A pll-B* 85% reduced length compared to TILLING control (Figure 5M).

For three-day-old seedlings grown under long-day conditions, the network width, depth, width to depth ratio, convex hull area, and angle of seminal roots in *ccd-A4 ccd-B4 pll-A pll-B* were largely decreased relative to TILLING control. These observed root phenotypes are similar to those observed for the dark-grown seedlings (Figures 6A–E). When compared to TILLING control, the total seminal root length was reduced by 80% in *ccd-A4 ccd-B4 pll-A pll-B* (containing only seminal roots 1–3), 31% in *ccd-A4 pll-A*, and 39% in *ccd-B4 pll-B* (Figures 6I–K). The coleoptile lengths of *ccd-A4 pll-A, ccd-B4 pll-B*, and *ccd-A4 ccd-B4 pll-A pll-B* were 30, 43, and 92% decreased relative to TILLING control, respectively (Figure 6K). Interestingly, the long-day-grown seedlings generally exhibited narrower network width, smaller convex hull area, and more shallow seminal root angles than dark-grown seedlings (Figures 5, 6).

## Discussion

In this study, we isolated, generated, and characterized tetraploid wheat TILLING mutants of *ccd-A4 pll-A, ccd-B4 pll-B*, and *ccd-A4 ccd-B4 pll-A pll-B*. The carotenoid content was comparable in grains of TILLING control and the mutants, indicating that CCD4 homoeologs do not play a major role in carotenoid turnover in grains, and therefore their activities do not need to be modified for provitamin A biofortification in tetraploid wheat grains. On the other hand, the moderately increased accumulation of neoxanthin in leaves of *ccd-B4 pll-B* and *ccd-A4 ccd-B4 pll-A pll-B*, violaxanthin in leaves of *ccd-A4 pll-A* and *ccd-B4 pll-B*, and lutein in leaves *ccd-B4 pll-B*, suggests a potential role of CCD-A4 and CCD-B4 in turnover of these xanthophylls in this tissue (Table 1). By contrast, β-carotene levels were not significantly changed in *ccd-A4 pll-A, ccd-B4 pll-B*, and *ccd-A4 ccd-B4 pll-A pll-B* relative to TILLING control in leaves and whole grains (Tables 1, 2), which is consistent with the results of *in vitro* enzyme assays where incubating recombinant CCD-A4 and CCD-B4 proteins with β-carotene did not yield any products (6). It should be noted that the catalytic activity of CCD-A4/CCD-B4 toward neoxanthin and violaxanthin was not examined in the enzyme assays previously (6).

Interestingly, four unidentified carotenoid peaks showed unique accumulation in leaves of *ccd-A4 ccd-B4 pll-A pll-B*, suggests that these carotenoid molecules could be potential substrates for CCD-A4 and CCD-B4 *in planta*. It also indicates that manipulation of CCD4 activities may lead to accumulation of carotenoids that are not normally present in wild-type plants (Figure 2; Supplementary Table 3). Furthermore, two carotenoids (peaks 3 and 4) are only present in *ccd-A4 ccd-B4 pll-A pll-B* leaves, suggesting that CCD-A4 and CCD-B4 may have overlapping activities toward these two carotenoids and can compensate for each other's missing activity in *ccd-A4 pll-A* and *ccd-B4 pll-B* leaves. This notion of overlapping activities is also supported by the observation that *CCD-A4* expression was induced when CCD-B4 function was abolished in *ccd-B4 pll-B* (Figure 3).

Besides analyzing the function of CCD4 homoeologs, the presence of *pll-A* and *pll-B* mutations in the TILLING mutants analyzed in this study also provides an opportunity for examining the role of PLL in wheat plants. Although six putative *PLL* genes were identified in the wheat genome, none of them have been functionally characterized (23). The *ccd-A4 pll-A, ccd-B4 pll-B*, and *ccd-A4 ccd-B4 pll-A pll-B* mutants displayed changes in several traits that contribute to wheat plant biomass and yield, including seminal root initiation and architecture, spike fertility, and grain size (Figures 4–6). These growth phenotypes have not been reported in the *ccd4* or *pol*/*pll* mutants characterized in *Arabidopsis* or other plant species. Conversely, the tetraploid wheat *ccd-A4 ccd-B4 pll-A pll-B* mutant does not exhibit the defective meristem and vasculature development phenotypes reported for the *Arabidopsis pol*/*pll* mutants (Figure 1).

Among the growth and agronomic traits altered by *ccd4* and/or *pll* mutations, mutations in the A or B subgenome homoeologs of *CCD4* and *PLL* alone already had a significant impact on the convex hull area of seminal roots, the seminal root length (1-3, 4-5), and the coleoptile length; these mutant phenotypes were exacerbated in *ccd-A4 ccd-B4 pll-A pll-B* where both A and B subgenome homoeologs were knocked out (Figures 5, 6). This suggests that the A and B subgenome homoeologs of CCD4 and/or PLL play distinct roles in controlling the above-mentioned traits. However, for other traits measured, such as number of spikelets per spike, grain yield, and seminal root initiation, significant differences were only observed in *ccd-A4 ccd-B4 pll-A pll-B*, suggesting that CCD4 and/or PLL homoeologs are functionally redundant for these traits.

Taken together, functional characterization of the *ccd-A4 pll-A, ccd-B4 pll-B*, and *ccd-A4 ccd-B4 pll-A pll-B* TILLING mutants uncovered the function of CCD4 in carotenoid accumulation in leaves and grains of tetraploid wheat. Additionally, the mutant analysis revealed that CCD4 and/or PLL homoeologs affect key seminal root, grain, and spike traits—traits that are important for not only wheat yield but also human nutrition as wheat grains are a critical dietary source of starch and proteins for human consumption. While the linked *ccd4* and *pll* mutations in the TILLING mutants pose challenges to discerning the role of CCD4, PLL or the interaction of CCD4 and PLL in controlling the plant growth traits, Clustered Regularly Interspaced Short Palindromic Repeats (CRISPR)/CRISPR-associated protein 9 (Cas9) (CRISPR/Cas9) gene editing lines of *CCD4* and *PLL* are currently being generated that mutate each gene individually. These CRISPR/Cas9-induced *ccd4* and *pll* mutant lines will help dissect the function of CCD4 and PLL in plant growth, which will have broad implications in improving wheat yield and nutrient content.

## Data Availability Statement

The original contributions presented in the study are included in the article/Supplementary Material, further inquiries can be directed to the corresponding author.

## Author Contributions

SY and LT designed the experiments, analyzed the data, and wrote the manuscript. SY performed the experiments. Both authors contributed to the article and approved the submitted version.

## Funding

This work was funded by USDA-NIFA (2017-67013-26164 to LT). SY received support from a China Scholarship Council Scholarship, the Henry A. Jastro Research Award, and the UC Davis, Department of Plant Sciences Graduate Research Fellowship.

## Conflict of Interest

The authors declare that the research was conducted in the absence of any commercial or financial relationships that could be construed as a potential conflict of interest.

## Publisher's Note

All claims expressed in this article are solely those of the authors and do not necessarily represent those of their affiliated organizations, or those of the publisher, the editors and the reviewers. Any product that may be evaluated in this article, or claim that may be made by its manufacturer, is not guaranteed or endorsed by the publisher.

## Supplementary Material

Supplementary Figure 1 Multiple sequence alignment of tetraploid wheat CCD-A4 and CCD-B4 as well as maize VP14 and *Synechocystis* sp. PCC 6803 ACO proteins. The conserved amino acids essential for carotenoid cleavage dioxygenase enzyme activities are indicated with asterisks. The mutated amino acids in CCD-A4 and CCD-B4 are indicated with blue and green arrows, respectively. VP14, 9-*cis*-epoxycarotenoid dioxygenase; ACO, lignostilbene dioxygenase.

Supplementary Figure 2 Multiple sequence alignment of tetraploid wheat PLL-A and PLL-B as well as *Arabidopsis* POL and PLL1 proteins. The metal interacting domains and conserved amino acids are underlined and indicated with asterisks, respectively. The mutated amino acids in PLL-A and PLL-B are pointed with blue and green arrows, respectively. POL, Poltergeist; PLL, Poltergeist-like.

Supplementary Figure 3 Images of 11-day-old *ccd-A4 ccd-B4 pll-A pll-B* and TILLING control seedlings grown in the dark. The white arrow points to the emerged seminal root 4 (SR4) in one of the 11-day-old *ccd-A4 ccd-B4 pll-A pll-B* seedlings. The white triangles indicate the places where root growth of TILLING control seedlings was constrained by the size of seed pouches.

Supplementary Table 1 Primers and restriction enzymes used in the genotyping analysis.

Supplementary Table 2 Primers used for amplification of the *PLL* homoeologs.

Supplementary Table 3 Areas (peak area mg^−1^ fresh weight) of peaks 1–4 integrated in the HPLC analysis shown in Figure 2B.
