# Supplementary figures and images for: Assessing the Role of Carotenoid Cleavage Dioxygenase 4 Homoeologs in Carotenoid Accumulation and Plant Growth in Tetraploid Wheat
Source: Front Nutr. 2021 Sep 8;8:740286. doi: 10.3389/fnut.2021.740286 (PMC8455956; doi:10.3389/fnut.2021.740286)

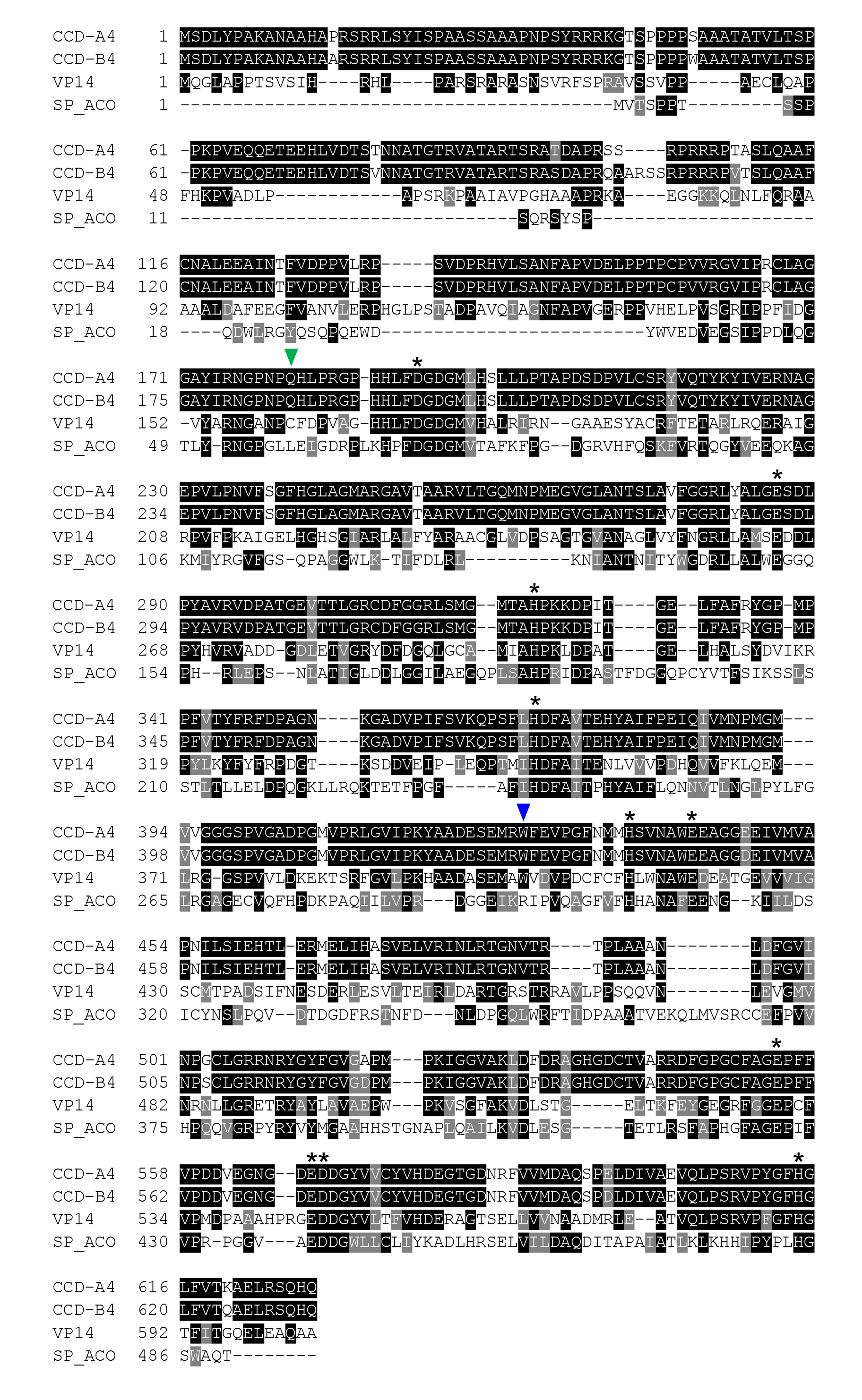

Supplement: Supplementary Figure 1 — Multiple sequence alignment of tetraploid wheat CCD-A4 and CCD-B4 as well as maize VP14 and Synechocystis sp. PCC 6803 ACO proteins. The conserved amino acids essential for carotenoid cleavage dioxygenase enzyme activities are indicated with asterisks. The mutated amino acids in CCD-A4 and CCD-B4 are indicated with blue and green arrows, respectively. VP14, 9-cis-epoxycarotenoid dioxygenase; ACO, lignostilbene dioxygenase. [file Image_1.TIF]

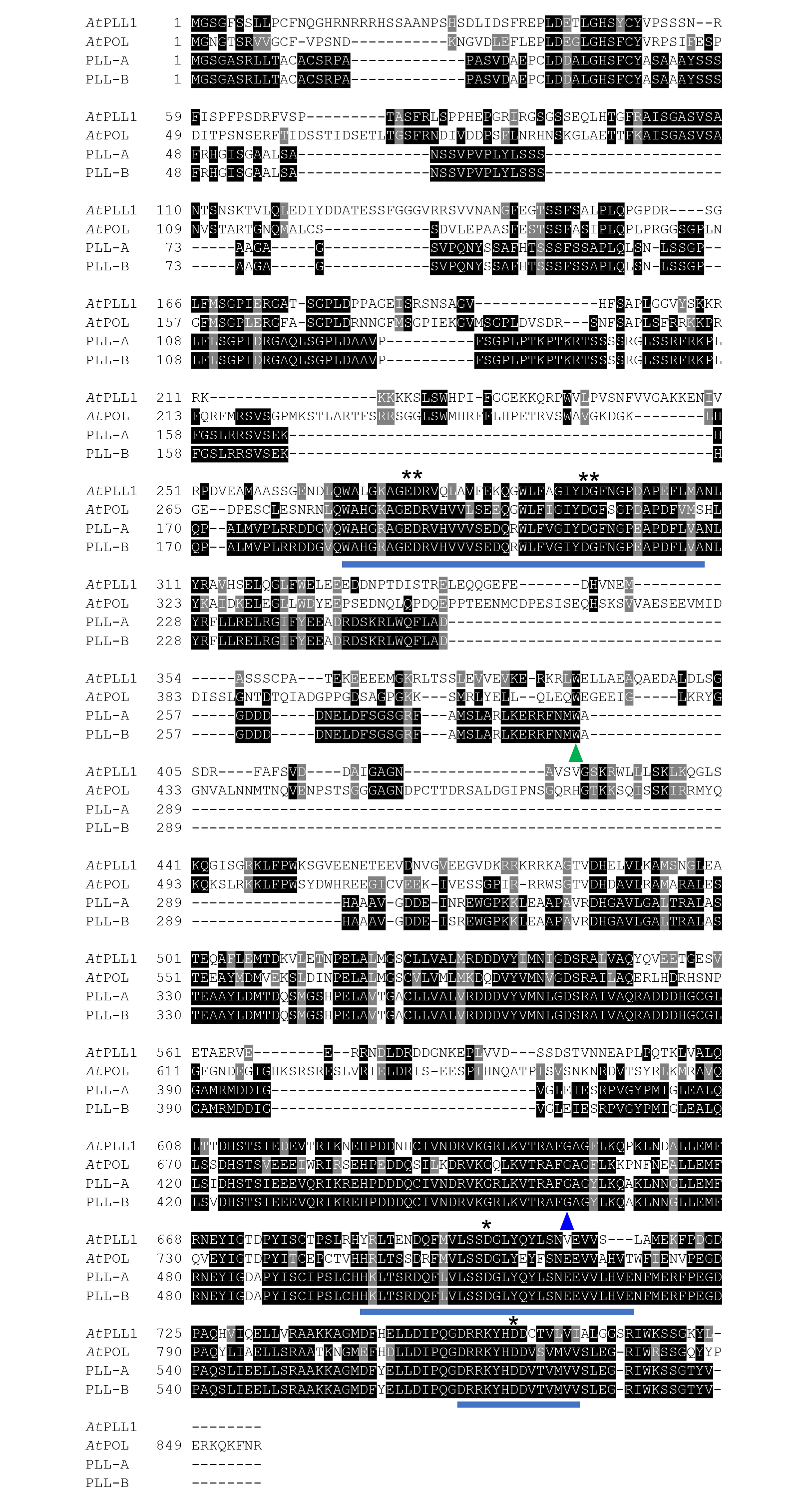

Supplement: Supplementary Figure 2 — Multiple sequence alignment of tetraploid wheat PLL-A and PLL-B as well as Arabidopsis POL and PLL1 proteins. The metal interacting domains and conserved amino acids are underlined and indicated with asterisks, respectively. The mutated amino acids in PLL-A and PLL-B are pointed with blue and green arrows, respectively. POL, Poltergeist; PLL, Poltergeist-like. [file Image_2.TIF]

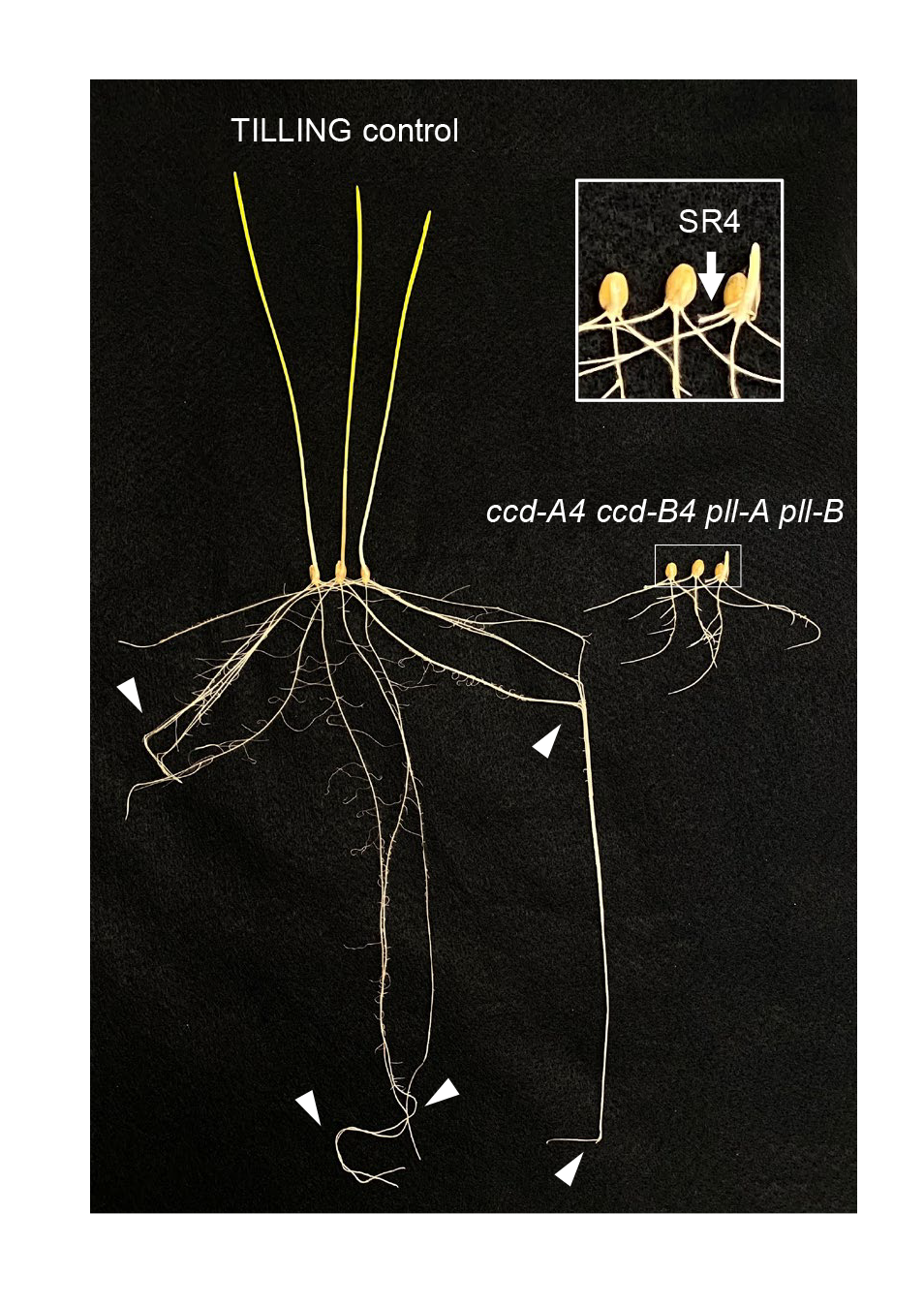

Supplement: Supplementary Figure 3 — Images of 11-day-old ccd-A4 ccd-B4 pll-A pll-B and TILLING control seedlings grown in the dark. The white arrow points to the emerged seminal root 4 (SR4) in one of the 11-day-old ccd-A4 ccd-B4 pll-A pll-B seedlings. The white triangles indicate the places where root growth of TILLING control seedlings was constrained by the size of seed pouches. [file Image_3.TIF]
