# Supplementary material for: Assessing the Role of Carotenoid Cleavage Dioxygenase 4 Homoeologs in Carotenoid Accumulation and Plant Growth in Tetraploid Wheat
Source: Front Nutr. 2021 Sep 8;8:740286. doi: 10.3389/fnut.2021.740286 (PMC8455956; doi:10.3389/fnut.2021.740286)
Supplement: Supplementary Table 1 — Primers and restriction enzymes used in the genotyping analysis. [file Table_1.DOCX]

**Table S1.** Primers and restriction enzymes used in the genotyping analysis. The mismatched nucleotide in the dCAPS marker is underlined. CAPS, cleaved amplified polymorphic sequence; dCAPS, derived CAPS; bp, base pair.

| Homoeolog | Primers | Amplicon (bp) | Marker/Restriction enzyme |
| --- | --- | --- | --- |
| *CCD-A4* | Forward: 5’-CCGACGTGCCCATCTTCT-3’  Reverse: 5’-AACATCTGGCAGTGGTGTG-3’ | 854 | CAPS/HphI |
| *CCD-B4* | Forward: 5’-CCTCGCCGCCGGCCCGTCA-3’  Reverse: 5’-CGGCCCGCGCGGGAGATGGT-3’ | 255 | dCAPS/RsaI |
| *LCYe-B* | Forward: 5’-TGAGAAGGTACATTCGATCTTG-3’ Reverse: 5’-TACAAGGTCAGGTAGGTCCTGA-3’ | 1773 | CAPS/DdeI |
