# Supplementary material for: Assessing the Role of Carotenoid Cleavage Dioxygenase 4 Homoeologs in Carotenoid Accumulation and Plant Growth in Tetraploid Wheat
Source: Front Nutr. 2021 Sep 8;8:740286. doi: 10.3389/fnut.2021.740286 (PMC8455956; doi:10.3389/fnut.2021.740286)
Supplement: Supplementary Table 2 — Primers used for amplification of the PLL homoeologs. [file Table_2.DOCX]

**Table S2.** Primers used for amplification of the *PLL* homoeologs.

| Homoeolog | Primers | Amplicon (bp) |
| --- | --- | --- |
| *PLL-A* | Forward: 5’- TTGTGGCATTGGTGAGGG -3’  Reverse: 5’- TAATATGTCAGGGACAGTCAATTT -3’ | 1675 |
| *PLL-B* | Forward: 5’- ACCTCTACCGCTTCCTCCTAC -3’  Reverse: 5’- CCAACCAAGCCGTACCACA -3’ | 1315 |
