# Supplementary material for: Assessing the Role of Carotenoid Cleavage Dioxygenase 4 Homoeologs in Carotenoid Accumulation and Plant Growth in Tetraploid Wheat
Source: Front Nutr. 2021 Sep 8;8:740286. doi: 10.3389/fnut.2021.740286 (PMC8455956; doi:10.3389/fnut.2021.740286)
Supplement: Supplementary Table 3 — Areas (peak area mg−1 fresh weight) of peaks 1–4 integrated in the HPLC analysis shown in Figure 2B. [file Table_3.DOCX]

**Table S3.** Areas of peaks 1-4 (peak area mg^-1^ fresh weight) integrated in the HPLC analysis shown in Figure 2B. Different letters denote significantly different (*P* < 0.05) areas for each peak. Peak 1 area of *ccd-A4 ccd-B4 pll-A pll-B* is marginally different (*P* = 0.0627) from that of TILING control.

|  | Peak 1 | Peak 2 | Peak 3 | Peak 4 |
| --- | --- | --- | --- | --- |
| TILLING control | 52.57 ± 8.95^a^ | 5.37 ± 0.48^a^ | ND | ND |
| *ccd-A4 pll-A* | 57.37 ± 15.33^a^ | 5.03 ± 0.09^a^ | ND | ND |
| *ccd-B4 pll-B* | 57.12 ± 22.62^a^ | 4.54 ± 0.36^a^ | ND | ND |
| *ccd-A4 ccd-B4 pll-A pll-B* | 37.33 ± 6.77^a^ | 13.56 ± 2.13^b^ | 24.91 ± 5.90 | 16.84 ± 3.19 |
